# Investigation of presenteeism, physical function, and exercise habits in workers with CKD: three case reports with literature review

**DOI:** 10.1186/s41100-022-00403-w

**Published:** 2022-04-13

**Authors:** Aki Tabata, Hiroki Yabe, Takehide Katogi, Tomoya Yamaguchi, Yuya Mitake, Tomohiro Shirai, Takayuki Fujii

**Affiliations:** 1 Department of Rehabilitation, Seirei Sakura Citizen Hospital, 2-36-2 Ebaradai, Sakura-shi, Chiba 285-8765 Japan; 2 Department of Physical Therapy School of Rehabilitation Science, Seirei Christopher University, 3453 Mikatahara, Kita-ku, Hamamatsu, Shizuoka 433-8558 Japan; 3 Department of Rehabilitation, Hamamatsu University Hospital, 431-3125, Hamamatsu, Shizuoka Japan; 4 Department of Nephrology, Seirei Sakura Citizen Hospital, 2-36-2 Ebaradai, Sakura-shi, Chiba 285-8765 Japan

**Keywords:** Chronic kidney disease, Presenteeism, Physical function

## Abstract

Management of presenteeism in the context of chronic kidney disease (CKD) is essential for disease management, ensuring the workforce’s availability, and reducing health-related costs. The purpose of this case study was to investigate presenteeism, physical function, and exercise habits in three working patients with CKD and discuss their effects. Case 1 was a 71-year-old male security guard; Case 2 was a 72-year-old male agricultural worker; and Case 3 was an 83-year-old male civil engineering employee. Presenteeism was measured using the work functioning impairment scale (WFun), and physical function was measured using grip strength, skeletal muscle mass index, 10 m walk test, short physical performance battery, and exercise habits. The WFun assessment showed that only Case 3 had moderate presenteeism, and the barrier to employment was fatigue. Each value of physical function was higher than the reference value, but Case 3 had the lowest physical function values. All three patients had no exercise habits and were in the interest stage of behavior change. This case report indicates the existence of workers with CKD who need care for presenteeism, even if they have no problems with physical function or activities of daily living. To ensure work productivity in workers with CKD, clinicians may need to evaluate presenteeism, physical function, and exercise habits in addition to popular treatment and care.

## Introduction

Presenteeism is defined as “people, despite complaints and ill health that should prompt rest and absence from work, still turning up at their jobs” [1]. Loss of productivity owing to presenteeism is a critical problem. The literature shows that presenteeism is associated with long-term sick leave [2], prolonged absence from work, and greater economic costs than those stemming from sick leave and sick days [3]. Furthermore, a previous study reported that the prevalence of work productivity loss in patients with non-dialysis chronic kidney disease (CKD) was 26.0%, of which absenteeism and presenteeism accounted for 7.1% and 22.3%, respectively [4]. The loss of work productivity accounts for a large proportion of the social costs in patients with CKD [5]. Managing presenteeism in the context of CKD is essential for disease management, ensuring the workforce’s availability, and reducing health-related costs. However, few studies have examined presenteeism among workers with CKD. Research has shown that lower physical function affects presenteeism [6] in healthy older workers. Additionally, lack of exercise habits or awareness may lead to a decline in physical function and indirectly affect presenteeism. However, presenteeism and physical function in working patients with CKD have not been thoroughly investigated.

The purpose of this case study was to investigate presenteeism, physical function, exercise habits, and self-efficacy (SE) of exercise in three working patients with CKD and discuss the effects of physical function and exercise status on presenteeism.

## Case report

The participants included three working patients with CKD from among the 16 patients with non-dialysis CKD admitted for CKD education at Seirei Sakura Citizen Hospital from August to October 2020. The characteristics of the three cases are listed in Table 1.

**Table 1:** Characteristics of each case

	Case 1	Case 2	Case 3
Age (years)	71	72	83
Sex	Male	Male	Male
Height (cm)	172.6	166.0	159.0
Weight (kg)	79.7	76.6	63.4
BMI (kg/m^2^)	26.8	27.8	25.1
Occupation	Security guard	Farmer	Civil engineering employee
Primary disease	Nephrosclerosis	Nephrosclerosis	IgA nephropathy
CKD stages	4	4	4
Laboratory parameters			
TP (g/dl)	7	6.6	5.7
Alb (g/dl)	3.6	3.9	3.3
BUN (mg/dl)	32	30	31
Cr (mg/dl)	1.9	2.7	2.0
eGFR (ml/min/1.73 m^2^)	28.9	18.9	25.7
Hb (g/dl)	12.8	10.7	10.7
Ht (%)	38.3	33.4	33.0
MCV (fL)	91.8	92.7	91.0
CRP (mg/dl)	0.0	0.1	0.1
Proteinuria (g/day)	0.8	1.0	1.1

*TP* total protein, *Alb* albumin, *BUN* blood urea nitrogen, *Cr* creatinine, *eGFR* estimated glomerular filtration rate, *Hb* hemoglobin, *Ht* hematocrit, *MCV* mean corpuscular volume, *CRP* C-reactive protein

### Case 1

A 71-year-old man with a height of 172.6 cm, weight of 79.7 kg, and body mass index (BMI) of 26.8. The patient was referred to our hospital with elevated creatinine levels and was admitted for CKD education. He lived at home with his wife and was independent in his activities of daily living (ADL). He was a security guard and worked as a company employee. The frequency of work was 3–4 days per week, including night shifts. At work, he mainly spent his time standing, and he also walked a lot. He had a past medical history of hypertension, hyperuricemia, dyslipidemia, and diabetes mellitus. The patient was taking olmesartan and febuxostat orally. A nurse, dietitian, and pharmacist guided his diet, medication, and daily life from the day after admission. A physical therapist evaluated presenteeism and physical function and managed exercise therapy on day 4 of admission. The physical therapist provided him with exercise guidance to help him incorporate exercise into his daily life while working. There were no significant changes in vital signs, weight, or urine output during hospitalization. The patient was discharged on the 12th day.

### Case 2

A 72-year-old man with a height of 166.0 cm, weight of 76.6 kg, and BMI of 27.8. The patient had been visiting our hospital as an outpatient for treatment of hypertension and CKD. There were no restrictions on his salt and alcohol intake at home, and his blood pressure was high. His creatinine level was elevated to 3.48 at the last outpatient visit. The patient lived alone at home and was independent in ADL. A self-employed agricultural worker, he had fewer than 3 days off per month and worked almost daily. At work, he mainly spent his time standing, and he also walked a lot. He had a past medical history of hypertension. The patient was taking febuxostat, nifedipine, prazosin hydrochloride, olmesartan, nifedipine CR tablets, and clonidine hydrochloride orally. Olmesartan, nifedipine CR tablets, and clonidine hydrochloride were administered on the day of admission. Nurses, dietitians, and pharmacists guided his diet, medication, and daily life from the day after admission. A physical therapist evaluated presenteeism and physical function and managed exercise therapy on day 5 of admission. The physical therapist provided him with exercise guidance to help him incorporate exercise into his daily life while working. His blood pressure fluctuated between 110 and 160 mmHg at the beginning of hospitalization but stabilized at 140 mmHg before discharge. The patient was discharged on the 9th day.

### Case 3

An 83-year-old man with a height of 159.0 cm, weight of 63.4 kg, and BMI of 25.1. The patient visited our hospital for IgA nephropathy. His renal function had been worsening since the previous year, so he was admitted for examination and CKD education. He lived at home with his wife and was independent in ADL. He was a civil engineer and worked as a company employee. The frequency of work was 5–6 days per week, and he spent most of that time standing and walking. He had a past medical history of hypertension, IgA nephropathy, hyperuricemia, and diabetes mellitus. The patient was taking olmesartan, epinastine hydrochloride, febuxostat, doxazosin mesylate, nifedipine, alogliptin benzoate, lansoprazole, and sennoside A, B. Nifedipine, alogliptin benzoate, lansoprazole, and sennosides A and B were started on the day of admission. A nurse, dietitian, and pharmacist guided his diet, medication, and daily life from the day after admission. A physical therapist evaluated presenteeism and physical function and managed exercise therapy on day 6 of admission. The physical therapist provided him with exercise guidance to help him incorporate exercise into his daily life while working. In addition, he was instructed in resistance training to improve muscle strength; this could be performed at home too. The patient was discharged on the 8th day.

## Measurements

Presenteeism was investigated using the work functioning impairment scale (WFun), a questionnaire developed at the University of Occupational and Environmental Health to objectively measure the degree of presenteeism due to health problems [7]. It consists of seven simple questions scored 1–5 points, for a total score of 7–35 points. WFun scores of 7–13 points indicate no problem, 14–20 points indicate mild disability, 21–27 points indicate moderate disability, and 28–35 points indicate severe disability. The higher the score, the higher the degree of presenteeism. In addition to the WFun, each patient was asked about barriers to employment. Grip strength, the 10 m walk test, short physical performance battery (SPPB), and skeletal muscle mass index (SMI) were measured to evaluate physical functions. The 10 m walk test was calculated from the number of seconds of comfortable walking in the 10 m section. The SPPB consists of three sub-items: a standing balance, walking, and chair-standing test scored 0–4 points each, for a total of 0–12 points. SMI was calculated from muscle mass measured using a multi-frequency bioelectrical impedance measurement device (Tanita MC-780) as the limb muscle mass divided by height squared. The results of the physical function assessment of each case were compared with the reference values of physical function from previous studies [8]: grip strength < 28 kg, SMI < 7.0 kg/m^2^, walking speed < 1.0 m/s, and SPPB ≤ 9. Exercise habits were investigated using the five stages of behavioral change (i.e., pre-contemplation, contemplation, preparation, action, and maintenance). Exercise SE was assessed using a partially modified version of Oka’s scale [9]. The score ranges from 4 to 20, and the higher the score, the higher the confidence in exercise. The scale was created in Japanese and has been demonstrated to be reliable and valid.

## Results

The results for each evaluation item are shown in Table 2. The WFun scores of Cases 1–3 were 7, 12, and 16, respectively, and only Case 3 had moderate presenteeism. Cases 1 and 2 did not experience any barriers to employment, but Case 3 did. He said, “I do much standing work, and I get tired quickly. I can no longer keep up with the pace of my subordinates work.” Handgrip strength (Case 1, 40.3 kg; Case 2, 39.6 kg; Case 3, 28.7 kg) was higher than the age-matched reference value (71 years old, 34.7 kg; 72 years old, 34.1 kg; 83 years old, 27.3 kg) [10]. The 10 m walk test value (Case 1, 1.12 m/s; Case 2, 1.18 m/s; Case 3, 1.07 m/s) was also higher than the age-matched reference value (70–79 years old, 1.02 m/s; ≥ 80 years old, 0.81 m/s) [11]. However, Case 3 had lower physical function values than Cases 1 and 2; additionally, there was only a slight difference when comparing Case 3 values to the reference value for age. SMI and SPPB were above the reference values [8] in all cases. All three cases were in the interest period of exercise habit, and exercise SE was low in Cases 2 and 3.

**Table 2:** Results of WFun, physical function, and exercise habits in each case

	Case 1 (71 years)	Case 2 (72 years)	Case 3 (83 years)	Reference values
WFun (score)	7	12	16	7–13 (no problem)
				14–20 (mild disability)
				21–27 (moderate disability)
				28–35 (severe disability) [7]
Grip strength (kg)	40.3	39.6	28.7	34.7 (71 years old)
				34.1 (72 years old)
				27.3 (83 years old) [10]
10 m walk test (m/s)	1.12	1.18	1.07	1.02 (70–79 years old)
				0.81 (≥ 80 years old) [11]
SMI (kg/m^2^)	9.16	9.87	8.30	< 7.0 [8]
SPPB (score)	12	12	11	⫹ 9 [8]
Exercise habits	No	No	No	
Stages of behavior change	Preaction	Preaction	Preaction	
Exercise SE (score)	14	8	5	

*WFun* Work Functioning Impairment Scale, *SMI* skeletal muscle mass index, *SPPB* short physical performance battery, *SE* self-efficacy

## Discussion

In this case study, Case 3 a worker with CKD had presenteeism, as measured by the WFun. To our knowledge, this is the first case study to demonstrate presenteeism in a worker with CKD despite their physical function not being below the standard value. These results indicate the existence of workers with CKD who appear to have no physical function or ADL problems but need care for presenteeism.

Presenteeism appears to have two main definitions [12]. “Sickness presenteeism” is defined as “people, despite complaints and ill health that should prompt rest and absence from work, still turning up at their jobs” [1]. “Impaired work function,” however, refers to “a reduced performance at work, besides illness” [13]. In this study, we used the former definition of presenteeism to include psychological and behavioral science views rather than just labor productivity. There are many scales that measure presenteeism: the Stanford Presenteeism Scale [14], Health and Work Performance Questionnaire [15], Work Limitation Questionnaire [16], Health and Work Questionnaire [17], Work Productivity and Activity Impairment Questionnaire (WPAI) [18], and the WFun. The WFun is a seven-item scale that is useful for easily assessing presenteeism in busy clinical settings; it is characterized by its presenteeism-specific assessment and its ability to identify the severity of presenteeism at four levels, compared to the other assessments. Furthermore, the WFun has been correlated with other standardized tools for measuring impaired work function [19] and validated consistent with Consensus-based Standards for the Selection of Health Measurement Instruments. However, the unification of the two definitions of presenteeism and the relationship between the above measurements to assess presenteeism are considered issues for the future.

Grip strength, SMI, 10 m walk test, and SPPB were the lowest in Case 3. A decline in physical activity has been reported as the main reason for stopping work among healthy older workers [20]. The physical function of Case 3 was not sufficient for him to work without experiencing fatigue, which may have affected his presenteeism. Consequently, each physical function value was above the standard value in Case 3. The amount of physical activity of workers differs depending on the type of job [21]. A job requiring much activity may need a higher level of physical function than the population value to maintain work productivity. In the present study, all three patients had jobs that required much physical activity, mainly standing and walking. Therefore, the reference values from previous studies may not be enough to detect a decline in physical function that leads to presenteeism. This requires further research.

The WFun results showed that only Case 3 had moderate presenteeism, and the barrier to employment was fatigue. In a previous study, fatigue was reported to be a disincentive to employment in workers since it was associated with presenteeism and absenteeism [22]. Moreover, it is a major symptom in patients with CKD, and subjective fatigue during exertion is reportedly stronger in this population than in healthy subjects [23]. Older patients with CKD are more likely to feel fatigued during work, leading to decreased work productivity. In Case 3, fatigue related to CKD may have affected presenteeism. A previous study showed that exercise therapy in patients with CKD reduces fatigue [24]. Additionally, physical function is related to fatigue in workers [25], and exercise therapy for workers has been shown to reduce fatigue [26]. Many factors affect fatigue in patients with CKD, but exercise and physical activity may be potentially beneficial factors. Management of exercise habits may be necessary for addiction to other treatments and care for CKD to improve fatigue and presenteeism.

All three cases were in the interest period of exercise habit, and exercise SE was low in Cases 2 and 3. Previous studies have reported that cardio training and major muscle strength training once a week within working hours [27] or short exercises (stretching, cognitive functional training, aerobic exercise, body weight resistance training, and cool-down) for 10 min a day, 3–4 times per week during lunch break [28], reduced presenteeism—suggesting that exercise therapy may contribute to the reduction in presenteeism. However, most patients with CKD have barriers to starting and continuing exercise. Exercise SE is a substantial factor in the continuation of exercise. The decline in SE may be a potential factor in presenteeism owing to a lack of exercise habits and reduced physical function. Improving exercise SE and continuing exercise in working patients with CKD may reduce fatigue, maintain and improve physical function, and contribute to the improvement of presenteeism. Therefore, to evaluate the working and exercise status of patients with CKD, exercise guidance is needed for them to exercise in their daily lives while improving their SE.

One of the limitations of this study is that it cannot examine the effects of exercise instruction. It is necessary to investigate the effect of exercise instruction on presenteeism in the future. In addition, the comparison of results when using different definitions of presenteeism and their relationship with other measurement methods and their validity must be addressed in the future.

This case report indicates the existence of workers with CKD requiring care for presenteeism, even if they have no physical function or ADL problems. In addition, we consider that screening using the WFun is important to detect presenteeism in working patients with CKD. To ensure work productivity in workers with CKD, clinicians may need to evaluate presenteeism, physical function, and exercise habits in addition to popular treatment and care.

## Literature review

To our knowledge, there are three previous studies on presenteeism in patients with pre-dialysis CKD (Table 3), all of which used the WPAI to assess presenteeism. In a report by van Haalen et al. [4], patients with CKD Stages 3–5 and dialysis were included. They reported that the prevalence of loss due to presenteeism was 18.8% for Stage 3a, 19.6% for Stage 3b, 27.9% for Stage 4, 21.3% for Stage 5, 22.3% for all pre-dialysis CKD patients, and 34.7% for dialysis. Furthermore, presenteeism was associated with hemoglobin levels. In the report by Eriksson et al. [29], patients with autosomal dominant polycystic kidney disease were included. They reported that the prevalence of loss due to presenteeism was 7.4% in CKD Stages 1–3 and 18.8% in CKD Stages 4–5. Covic et al. [30] investigated the effects of cardiovascular disease and anemia on occupational dysfunction in patients with CKD Stages 3–4. The presence of cardiovascular conditions was associated with work productivity and activity impairment, particularly among patients with anemia. However, it was not associated with presenteeism. Previous studies investigating presenteeism in patients with pre-dialysis CKD are limited. The rate of presenteeism loss in CKD patients is high, and it increases as the CKD stage progresses, with dialysis patients having a higher rate compared to pre-dialysis CKD patients. Furthermore, hemoglobin levels have been reported as a factor affecting presenteeism. However, there is a lack of prospective studies on the course of presenteeism in CKD patients, and the impact of physical function on presenteeism in pre-dialysis CKD patients has not been investigated.

**Table 3:** Previous studies on presenteeism in pre-dialysis CKD patients

Study	Subject	Measures of presenteeism	Percentage of loss due to presenteeism	Main results
Haalen et al. [4]	CKD stages 3–5	WPAI	CKD stage 3a: 18.8%	Lower hemoglobin levels worsen the impact of CKD on QOL, and are associated with lower work productivity in patients with CKD
			CKD stage 3b: 19.6%	
			CKD stage 4: 27.9%	
			CKD stage 5: 21.3%	
			All CKD stage: 22.3%	
Eriksson et al. [29]	CKD stages 1–5 (ADPKD)	WPAI	CKD stages 1–3: 7.4%	Mean total direct and indirect costs were approximately twice as high in patients with CKD stages 4–5 compared to CKD stages 1–3
			CKD stages 4–5: 18.8%	
Covic et al. [30]	CKD stages 3–4	WPAI	N/A	The presence of concomitant cardiovascular conditions was more common in non-dialysis CKD patients with comorbid anemia, and was associated with reduced QOL and work productivity outcomes

*CKD* chronic kidney disease, *WPAI* work productivity and activity impairment, *ADPKD* autosomal dominant polycystic kidney disease, *QOL* quality of life

In recent years, the impact of the COVID-19 pandemic on presenteeism has become a major issue; some previous studies have reported that the COVID-19 pandemic may have increased presenteeism [31–34]. Telecommuting not only blurs the distinction between work and personal life but also makes it harder for managers to monitor the health of their employees. In some cases, lack of access to medical resources [35] and increased treatment interruption [36] may exacerbate chronic diseases. This may consequently lead to an increased prevalence of presenteeism in CKD patients compared to that before the COVID-19 pandemic.

## Data Availability

All data in this case are available upon request.

## Abbreviations

CKD: Chronic kidney disease
WFun: Work functioning impairment scale
ADL: Activities of daily living
SMI: Skeletal muscle mass index
SPPB: Short physical performance battery
SE: Self-efficacy
TP: Total protein
Alb: Albumin
Cr: Creatinine
BUN: Blood urea nitrogen
eGFR: Estimated glomerular filtration rate
Hb: Hemoglobin
Ht: Hematocrit
MCV: Mean corpuscular volume
CRP: C-reactive protein
WPAI: Work productivity and activity impairment questionnaire
